# Effect of Off-Axis Ply on Tensile Properties of [0/θ]_ns_ Thin Ply Laminates by Experiments and Numerical Method

**DOI:** 10.3390/polym13111809

**Published:** 2021-05-31

**Authors:** Junfeng Hu, Xi Deng, Xutong Zhang, Wen-Xue Wang, Terutake Matsubara

**Affiliations:** 1 School of Mechanical and Power Engineering, Nanjing Tech University, No. 30 Pu Zhu South Road, Nanjing 211816, China; zhxtchina@163.com; 2 School of Materials Science and Engineering, South China University of Technology, No. 381 Wushan Road, Tianhe District, Guangzhou 510641, China; 3 Research Institute for Applied Mechanics, Kyushu University, Kasuga 816-8580, Japan; bungaku@riam.kyushu-u.ac.jp (W.-X.W.); tmatsuba@riam.kyushu-u.ac.jp (T.M.)

**Keywords:** thin ply laminate, off-axis ply, tensile test, crack behavior, finite element method

## Abstract

The effect of off-axis ply on the tensile properties of unbalanced symmetric [0/θ]_ns_ laminates was explored through experimental and numerical analysis. Six CFRP [0/θ]_2s_ plies with different off-axis angles θ were fabricated for tensile tests. In situ observations of the damage to the laminates were conducted to investigate the initiation and progressive growth of the laminates during the tension tests. The fiber fractures, crack initiation, and progressive propagation were analyzed by observing the free edge of the laminates, and the difference in damage behavior caused by different off-axis angles was investigated. All the six [0/θ]_2s_ plies with off-axis angles θ ranging from 15° to 90° showed approximate linear stress–strain responses in the tensile tests. Matrix cracks were not observed prior to the final catastrophic failure in the off-axis layers of the [0/θ]_2s_ laminates with a θ in the range of 15–60°. Finite element analysis (FEA) of the [0/θ]_s_ plies was conducted using a 3D micromechanical model, in which matrix cracking and fiber-matrix debonding in the off-axis layer were simulated using a cohesive interface element. Three micromechanical crack-free, cohesive interface, and initial crack models were analyzed to predict the influence of the matrix cracks inside the off-axis layer on the damage behavior of the [0/θ]_s_ laminates. The numerical results from the initial crack micromechanical model show a lower bound of the tensile strength of the [0/θ]_s_ plies. A high stress concentration is observed adjacent to the cracked off-axis layer, inducing a tensile strength loss of about 20%.

## 1. Introduction

Carbon fiber reinforced polymer (CFRP) laminates have been attracting much attention due to their outstanding mechanical properties in the fiber orientation direction. Multidirectional laminates consisting of two or more angles of off-axis layers are able to improve mechanical properties in multiple directions. However, the failure mechanism of such multidirectional laminates with an axis layer and multiple off-axis layers may become much more complicated than unidirectional (UD) laminates. The initiation and growth of matrix cracks parallel to the fiber orientation in off-axis plies are always observed. Moreover, matrix cracks easily cause the initiation of other damage events, such as delamination, matrix cracks, and fiber fractures in neighboring ply. Thus, the investigation of the effect of the microscopic damage in the off-axis layers on the mechanical properties of multidirectional laminates is necessary for accurately predicting the mechanical properties of multidirectional laminates subjected to an external load. Many experimental and numerical efforts have been made to analyze the effect of matrix cracks on the mechanical properties of various multidirectional laminates [1,2,3,4,5,6].

In general, matrix cracks degrade the mechanical properties of composites and induce other types of damage, finally leading to the fracturing of the whole laminate. Matrix cracks can form in any plies, but they always initiate in off-axis layers and then propagate towards the fiber orientation [7,8]. Cross-ply laminate is a special type of multidirectional laminate, and transverse matrix cracks in 90° plies are easily observed in tensile tests. Many efforts have been conducted to investigate the failure mechanism for various cross-ply laminates [9,10,11,12]. Takeda and Ogihara [9] measured the delamination ignition and crack growth of transverse cracks in CFRP cross-ply laminates using loading/unloading tests, and Groves et al. [10] investigated two types of crack initiation and propagation in cross-ply laminates experimentally and numerically. Moreover, Smith et al. compared the transverse cracking behavior in surface plies of CFRP laminates ([90/0]_s_ and [0/90]_s_) and discussed the role of defects in the crack initiation [11]. Their research results demonstrated that increasing the thickness of the 90° layer reduced the stiffness of the laminates and led to more curved matrix cracks, while reducing the matrix crack density. The cross-ply laminate is one type of laminates with [0_n_/θ_m_]_s_. Pakdel and Mohammadi [12] developed an energy-based criteria, which can be used to predict the growth of matrix cracks in laminates of [0_n_/θ_m_]_s_. Their research indicated that a thicker off-axis layer had a lower strain and failed at a lower crack density and that a larger off-axis angle in the off-axis layers led to a lower strain and higher crack density. Some researchers focused on the effect of fiber rotations in angled-ply laminates in mitigating the inherent limitation of brittle failures of CFRP composites [13,14,15]. For instance, Fuller and Wisnom [13] investigated the non-linear stress–strain behavior of angled-ply laminates ([±5]_s_) with angles between 15° and 45° using thin ply carbon prepreg. Significant fiber rotations were observed due to the matrix plasticity, and the delamination was suppressed. Thomas et al. [14] achieved strain hardening behavior in angled laminates of [±45_n_]_s_ by fiber rotation, owing to an amorphous thermoplastic matrix.

In addition, theoretical and computational analyses, together with experimental research, are also useful and powerful tools for discovering the failure mechanism of various laminates. Kashtalyan and Soutis [16] predicted the strength loss attributed to matrix cracks in off-axis layers using a theoretical modeling of unbalanced symmetric laminates based on 2D shear lag theory. Vaughan and McCarthy [17] introduced a 2D micromechanical model to simulate the failure mechanism of UD CFRP laminate under a transverse tensile load. They investigated the influence of the properties of the fiber/matrix interface on the transverse strength and interfacial fracture toughness. Barulich et al. [18] investigated the effect of non-uniformly transverse matrix cracks in different cross-ply laminates of [0_n_/90_8_]_S_ and [90_8_/0_n_]_S_ using a computational meso-mechanical model. Their research results indicated that the cracks in a non-uniformly distributed meso-mechanical model agreed better with the experimental data than the cracks in a uniformly distributed model. Zhang and Herrmann [19,20] and Katerelos at al. [21] investigated the stiffness loss of various multidirectional laminates theoretically, numerically and experimentally. Their research results showed a good agreement with the experimental results. An analytical modelling method composed of matrix plasticity, fiber reorientation, and the classical laminate analysis for predicting the in-plane response of thin angle-ply laminates was proposed by Fuller and Wisnom [22], and it can demonstrate the non-linearity of angled-ply laminates with angles between 15°and 45°. While many studies on the effect of off-axis layers on the mechanical properties of various multidirectional laminates have been carried out, as mentioned above, most of the studies used laminates with a thicker off-axis layer than the 0° layer to achieve the easy observation of damage in the off-axis layer. However, in the practical application of composite laminate, it is often seen that each off-axis layer has an identical thickness in each 0° layer in a laminate, except for the central layer of a symmetrical laminate.

The present study focused on the effect of off-axis layer on tensile properties, including the strength and microscopic damage propagation of unbalanced symmetric [0/θ]_2s_ laminates using an in situ observation experiment and 3D micromechanical model analysis. Tensile tests with six CFRP [0/θ]_2s_ laminates were conducted. In situ observations of the progressive microscopic damage in the laminates were conducted to investigate the initiation and progressive growth of the laminates during the tension test. Damage events were observed on the free edge of six laminates. The microscopic damage in the central region in the width direction of the [0/θ]_2s_ laminate was also investigated. Numerical analysis of the [0/θ]_2s_ laminate was conducted using the 3D micromechanical model that was developed in the previous section. The potential matrix crack in the off-axis layer was simulated using an interface element with proper cohesive properties (in the matrix and at the matrix/fiber interface). Three matrix crack models were analyzed to predict the effect of the off-axis layer on the failure behavior of the [0/θ]_s_ laminates. This research revealed the damage mechanism for unbalanced symmetric [0/θ]_2s_ laminates through experimental and numerical analysis, and provided technical support for the design and application of CFRP composites with off-axis layers.

## 2. Experimental Procedure

### 2.1. Materials

**Figure 1:**
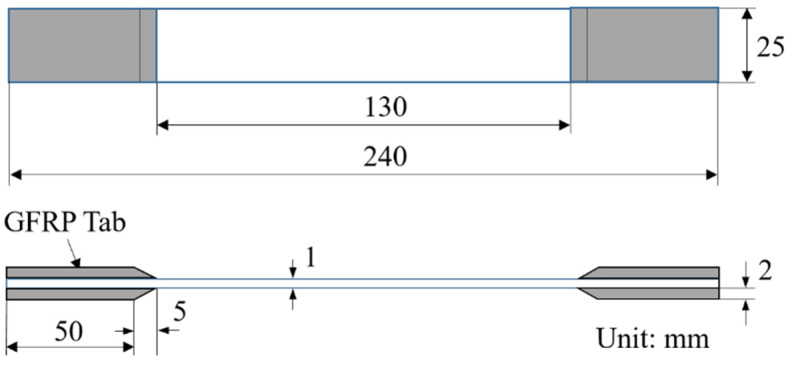
Dimensions of the tensile specimen of the [0/θ]_2s_ laminates.

The commercially available unidirectional CFRP prepreg of T700SC/#2592 (Toray industries, Inc. [23]) was used for the fabrication of the [0/θ]_2s_ laminates. The thickness of the prepreg is t_0_ = 0.12 mm, and the fiber volume fraction is about 58%. Referring to previous experimental data [24,25,26], the mechanical constants of unidirectional lamina used in the present study are listed in Table 1. Six unbalanced angle-ply [0/θ]_2s_ laminates in which the off-axis angles θ were 15°, 30°, 45°, 60°, 75°, and 90°, were fabricated using the autoclave cure method. The dimensions of the specimens for the tensile tests were 240 mm in the longitudinal direction and 25 mm in the width direction, following the standard ASTM D 3039, as illustrated in Figure 1. Glass fiber reinforced polymer (GFRP) tabs ([45/-45]_2s_) were pasted near the ends of the specimen. The GFRP tabs were 55 mm in length, 25 mm in width, and 2 mm in thickness. A strain gauge was set at the center of the specimen to test the tensile stress–strain relation. Five specimens were used for the evaluation of the tensile strength of each [0/θ]_2s_ laminate. Furthermore, three specimens were fabricated for the in-situ observation test of each [0/θ]_2s_ laminate.

### 2.2. Tensile Strength Test

The MTS-810 material testing system was used to conduct the tensile tests. A crosshead speed of 1 mm/min was used for all the tested specimens at an ambient temperature. The tensile strength of each type of [0/θ]_2s_ laminate was evaluated by the average of five specimens. The global failure behaviors of the tested specimens were observed at the macroscale using a real-time high-speed digital video (Sony RX100). However, the details of the initiation and propagation process at the microscopic scale were not observed in this step.

### 2.3. In Situ Microscale Observation of Microscopic Failure Process

A digital microscope of Keyence VHX-2000 and zoom lens of Keyence VH-Z50L were used to observe the failure process on the free edge of each specimen in situ during the tensile test. Real time continuous monitoring at the central point of the observation area was carried out during the tensile loading process, and detailed microscopic observation of the damage configuration on the observation area was conducted at three selected strength levels of 30%, 50%, and 80% at the averaged tensile strength of the laminate without unloading. A high speed digital camera was also used to record the macroscopic failure process.

## 3. Experimental Results

### 3.1. Results of Tensile Tests

**Figure 2:**
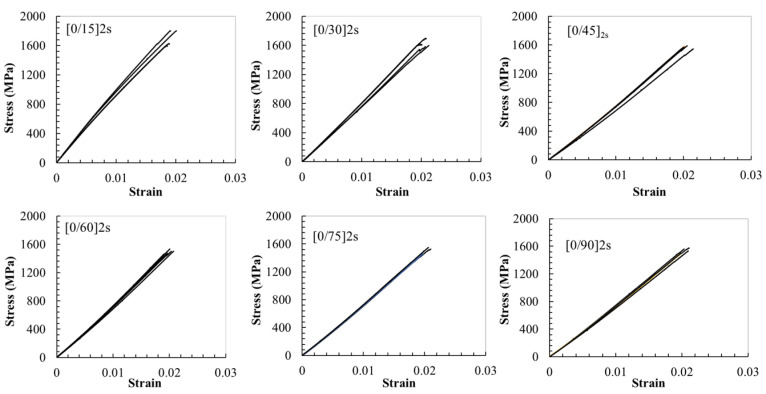
Stress–strain responses of various [0/θ]_2s_ laminates, and different lines in each subfigure represent different specimens of each [0/θ]_2s_ laminate.

The stress–strain responses of six [0/θ]_2s_ laminates (θ = 15°, 30°, 45°, 60°, 75°, and 90°) were investigated. All the stress–strain curves of the six [0/θ]_2s_ laminates exhibit an approximately linear behavior, until the final failure during the tensile test, as shown in Figure 2, indicating that the damage in the off-axis plies almost has no obvious effect on the total stress–strain curve of the [0/θ]_2s_ laminates. The different lines in each subfigure represent different specimens of each [0/θ]2s laminate. Detailed results of the tensile modulus, tensile failure strain, and tensile strength are listed in Table 2. From Table 2, it can be seen that the experimental accuracy of all the results is acceptable. The coefficients of variation (C.V.) of testing results are in the range of 1.1–4.2% for the tensile modulus, 1.9–3.8% for tensile failure strain, and 1.2–6.3% for tensile strength.

**Figure 3:**
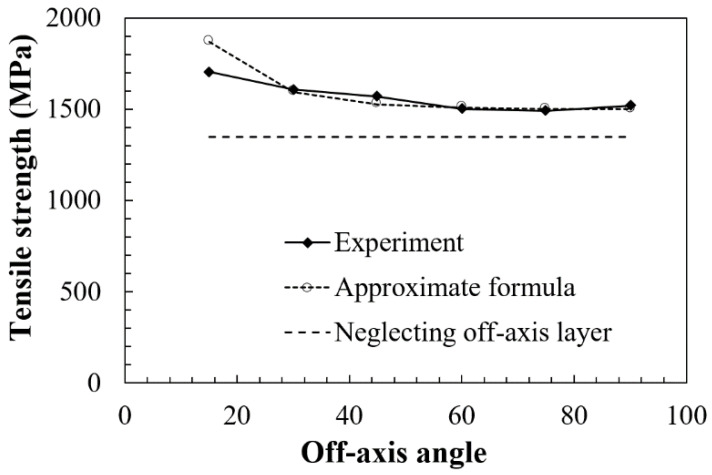
Relation of the failure strength of the [0/θ]2s laminate with an off-axis angle.

The strength of the [0/θ]_2s_ laminates varying with the off-axis angle is presented in Figure 3 together with an approximate theoretical curve and a reference stress value. The solid curve shows the experimental results. The horizontal dashed line denotes a reference stress that neglects the contribution of the off-axis ply to the laminate tensile strength, supposing that the UD layers in the [0/θ]_2s_ laminates bear all the tensile load, hence the stress does not vary with the off-axis angle for different [0/θ]_2s_ laminates. The dashed line with circle marks denotes the calculation results based on an approximate formula which is proposed in the present study for the calculation of the tensile strength of various multidirectional laminates with both 0° layers and θ off-axis layers. The formula was derived based on the classic lamination theory and the rule of mixture.

According to the classical lamination theory [27], the in-plane off-axis stiffness of a unidirectional lamina is easily calculated using the in-plane stiffness of a unidirectional ply. The off-axis stiffness constants ${\bar{S}}_{ij}$ (i, j=1, 2, 6) of one lamina can be calculated using the following equations:

(1) $$\bar{S_{11}}=\frac{1}{E_{x}}=\frac{1}{E_{1}}cos^{4}\theta +\left(\frac{1}{G_{12}}-\frac{2\nu_{12}}{E_{1}}\right)cos^{2}\theta sin^{2}\theta +\frac{1}{E_{2}}sin^{4}\theta$$

(2) $$\bar{S_{22}}=\frac{1}{E_{y}}=\frac{1}{E_{1}}sin^{4}\theta +\left(\frac{1}{G_{12}}-\frac{2\nu_{12}}{E_{1}}\right)cos^{2}\theta sin^{2}\theta +\frac{1}{E_{2}}cos^{4}\theta$$

(3) $$\bar{S_{66}}=\frac{1}{G_{xy}}=2\left(\frac{2}{E_{1}}+\frac{2}{E_{2}}+\frac{4\nu_{12}}{E_{1}}-\frac{1}{G_{12}}\right)sin^{2}\theta cos^{2}\theta +\frac{1}{G_{12}}\left(sin^{4}\theta +cos^{4}\theta \right)$$

where $E_{1}$, $E_{2}$, and $G_{12}$ are the in-plane longitudinal modulus, transverse modulus, and shear modulus of the UD lamina, respectively. $\nu_{12}$ represents the Poisson’s ratio. θ is the off-axis angle. $E_{x},\;E_{y}$, and $G_{xy}$ represent the off-axis tensile modulus, transverse modulus, and shear modulus of the UD lamina, respectively. Equations (1)–(3) can be used to predict the linear stiffness constants of an off-axis laminate with an arbitrary off-axis angle.

On the other hand, referring to the data on T700SC carbon fiber, epoxy resin, and Table 1, we can obtain the fiber failure strain $\varepsilon_{f}=2\%$ and the unidirectional tensile modulus $E_{1}=134\;\mathrm{GPa}$. As is well known, the [0/θ]_2s_ laminate under tension fails when the tensile strain reaches the fiber failure strain, because the tensile strength is dominated by the fiber strength of the 0° layer. Then, the tensile stresses of the 0° layer and off-axis layer in the [0/θ]_2s_ laminate at the tensile failure can be calculated as follows:

(4) $$\sigma_{0\_\mathrm{layer}}=E_{1}\times \varepsilon_{f},\;\sigma_{\theta \_\mathrm{layer}}=E_{x}\left(\theta \right)\times \varepsilon_{f}$$

Therefore, the tensile strength $\sigma_{\mathrm{laminate}}$ of the [0/θ]_2s_ laminate can be calculated as follows:

(5) $$\sigma_{\mathrm{laminate}}=\varepsilon_{f}\times [E_{1}\times V_{0\_\mathrm{layer}}+E_{x}\left(\theta \right)\times V_{\theta {\_}_{\mathrm{layer}}}]$$

based on the rule of mixture, where the $V_{0\_\mathrm{layer}}$ and $V_{\theta_{\mathrm{layer}}}$ are the volume fractions of the 0° layer and off-axis layer in the laminate, respectively, and $E_{x}\left(\theta \right)$ can be obtained from Equation (1). In the present case of the [0/θ]_2s_ laminates, both $V_{0\_\mathrm{layer}}$ and $V_{\theta_{\mathrm{layer}}}$ are 0.5. The calculation results of the tensile strength of the present [0/θ]_2s_ laminates are demonstrated by a dashed line with circle marks (approximate formula) in Figure 2. It is noted that the present approximate formula (5) generally gives the upper bound of the tensile strength of the multidirectional laminate because the effects of various damage events on the tensile strength of the laminate, such as matrix cracks, debonding and delamination, are not considered in this formula. The effect of matrix cracks on the [0/θ]_2s_ laminates will be analyzed later in this study.

Referring to Figure 3, it can be seen that the tensile strength of the [0/θ]_2s_ laminates exhibits an obvious reduction until θ = 60°, but has slight variation when θ > 60°. All the experimental results of the tensile strength in the range of 15°≤ θ ≤ 90° are larger than the reference stress (dashed line), which neglects the contribution of off-axis plies to the strength of the total laminate. These results reveal that off-axis plies still have a positive contribution to the total tensile strength. In addition, the calculated values of the tensile strength show a good consistency with the experimental results, suggesting that the damage in the [0/θ]_2s_ laminate has a significant influence on the tensile strength of the [0/θ]_2s_ plies when the θ is small. Additionally, the damage in the [0/θ]_2s_ plies with a relatively large θ has a slight influence on the tensile strength of the laminate.

### 3.2. Results of Microscopy Observation of Various Damage Events

**Figure 4:**
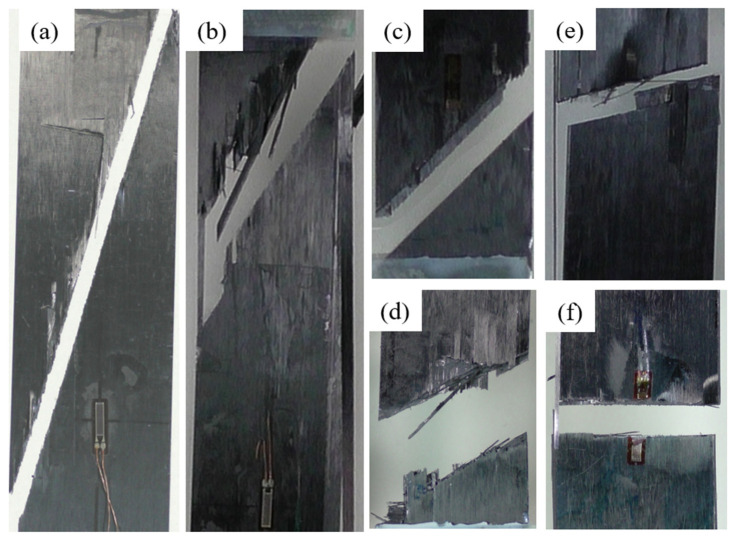
Failure images of the [0/θ]_2s_ laminate, (**a**) [0/15]_2s_, (**b**) [0/30]_2s_.(**c**) [0/45]_2s_, (**d**) [0/60]_2s_, (**e**) [0/75]_2s_, and (**f**) [0/90]_2s_.

Typical images of failed specimens of six [0/θ]_2s_ laminates are shown in Figure 4. In all the [0/θ]_2s_ laminates, the final fracture of each specimen always occurred along the corresponding off-axis angle of the [0/θ]_2s_ laminate, indicating that the initiation, accumulation, and propagation of matrix cracks in the off-axis layer have significant influence on the failure mechanism although the final failure is dominated by the 0° layer of the [0/θ]_2s_ laminate. The failure modes near the fracture section of each specimen are quite complicated. Fiber breakages in the 0° plies, matrix cracks in the off-axis plies, and delamination between the 0° and off-axis plies are observed near the fracture surfaces of each specimen. It is supposed that the matrix cracks in the off-axis layer propagate along the fiber direction, inducing the tensile stress concentration in the 0° layer and the interface between the 0° and off-axis plies in the vicinity of the crack tip, leading to the fracture of the 0° plies and delamination.

**Figure 5:**
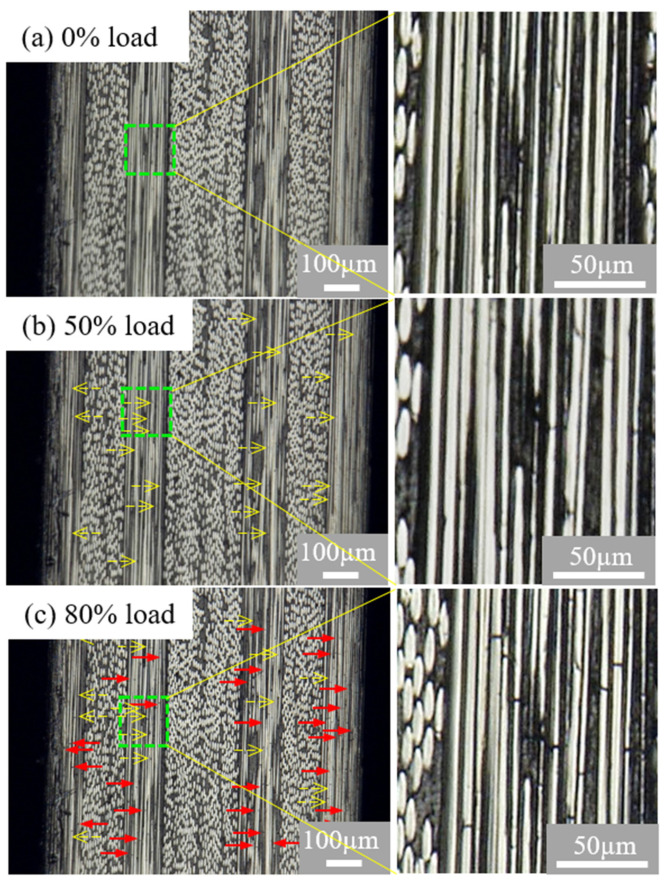
Damage images at the free edge of the [0/15]_2s_ laminates. (**a**) Before the test, (**b**) at 50% load, and (**c**) at 80% load. The dashed open arrows (yellow) refer to fiber breakages observed at 50% load; solid arrows (red) refer to new fiber breakages observed at 80%. The enlarged images are the green dashed regions.

Typical observations before the tensile test and at stress levels of 30%, 50%, and 80% of the failure stress were conducted for six types of [0/θ]_2s_ laminates. All the images were taken at the central 4-mm-long area of the polished free edge of the [0/θ]_2s_ laminates. The microscopic observation results for all the specimens at the 30% load level are omitted because no damage was observed in situ at this load level. The observation of one of the [0/15]_2s_ specimens is presented in Figure 5. In the image of the [0/15]_2s_ laminate before the tensile test, no matrix crack is observed at the polished free edge. When the tensile load increases to a strength level of 50% (tensile strain = 0.95%), a few fiber breakages marked by yellow arrows are observed in the surface areas of the 0° plies, but still no matrix crack is observed in the off-axis laminae. According to the material data of carbon fiber, the failure strain of carbon fiber is about 2%, and few fibers in the 0° plies generally break at such low tensile strain level, although fibers can break at any strain level. Hence, it is clearly possible that a few fiber breakages will be seen at the edges due to the edge effect. In fact, fiber breakages were only observed on the polished surface of the free edge, and no such fiber breakages were observed in the interior of the 0° plies based on the investigation of the damage progression in the width direction. As the tensile load further increases to the load level of 80% (tensile strain = 1.53%), many new fiber breakages marked by red arrows are observed, but still no obvious matrix cracks and delamination are observed prior to the final failure of the [0/15]_2s_ specimen. Similar images are observed on the free edge surface of the [0/30]_2s_, [0/45]_2s_, and [0/60]_2s_ laminates. Fiber breakages are the only damage events observed in the specimens of these three types of [0/θ]_2s_ laminates prior to the final failure of the specimens. These images tell us that the off-axis layers with θ < 60° have high resistance to matrix cracking. Matrix cracks and delamination observed on the fracture surfaces of each tested specimen are considered to occur close to or at the final catastrophic failure of the specimens. To capture the details of the super-high-speed final catastrophic failure process, a super-high-speed digital video camera measurement system seems to be required.

**Figure 6:**
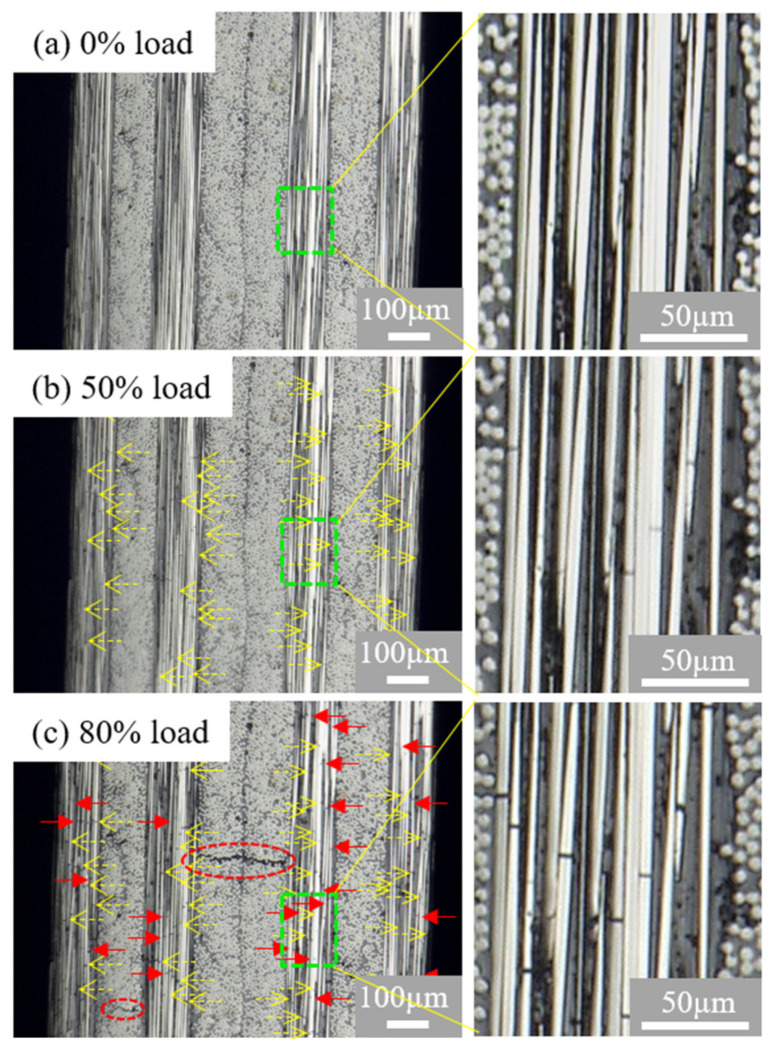
Failure images at the free edge of the [0/75]_2s_ laminates. (**a**) Before the test, (**b**) at 50% load, (**c**) at 80% load. The dashed open arrows (yellow) refer to the fiber breakages observed at 50% load; the solid arrows (red) refer to new fiber breakages observed at 80%. The red dash circle refers to matrix crack observed at 80% load. The enlarged images are the green dashed regions.

In contrast, different damage events are observed in the [0/75]_2s_ and [0/90]_2s_ laminates when the stress level increases to the 80% level, although there are still only fiber breakages observed in the [0/75]_2s_ and [0/90]_2s_ plies at the strength level of 50%. The observation of one of the [0/75]_2s_ specimens is presented in Figure 6. Matrix cracks can be seen in the off-axis plies of the [0/75]_2s_ laminates at the load level of 80% (tensile strain = 1.6%). Similar damage events are observed in the [0/90]_2s_ laminate as those in the [0/75]_2s_ specimens, except more matrix cracks are obtained. However, still no any delamination is observed in specimens of the [0/75]_2s_ and [0/90]_2s_ laminates prior to the final failure although many transverse matrix cracks are observed. The delamination is observed to occur close to or at the time of the final catastrophic failure of the specimen, and the details of the catastrophic failure process cannot be captured using the present digital camera measurement system. Further investigation is necessary to clarify this issue using super-high-speed digital video camera measurements if possible.

**Figure 7:**
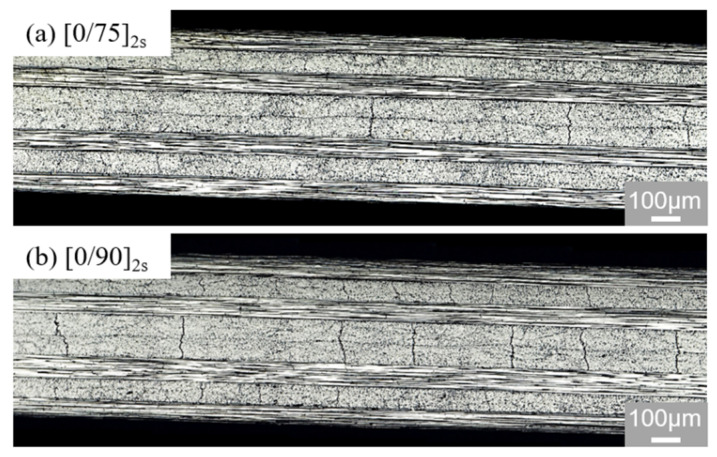
Images of the matrix cracks in the off-axis plies of the [0/75]_2s_ and [0/90]_2s_ laminates at the load level of 80%: (**a**) [0/75]_2s_ laminate; (**b**) [0/90]_2s_ laminate.

Typical images of matrix cracks in the central 2-mm-long area of the off-axis plies of the [0/75]_2s_ and [0/90]_2s_ specimens at the strength level of 80% are presented in Figure 7. Most of the matrix cracks spread approximately perpendicularly to the tensile direction. The matrix crack density increased with the tensile strain increasing once the matrix cracking initiated in the off-axis layers. The matrix cracking amount observed in the [0/90]_2s_ specimen was higher than that in the [0/75]_2s_ one. This result indicates that the cross-ply laminate (θ = 90°) has the lowest resistance to the initiation and accumulation of matrix crack among all the [0/θ]_2s_ laminates.

**Figure 8:**
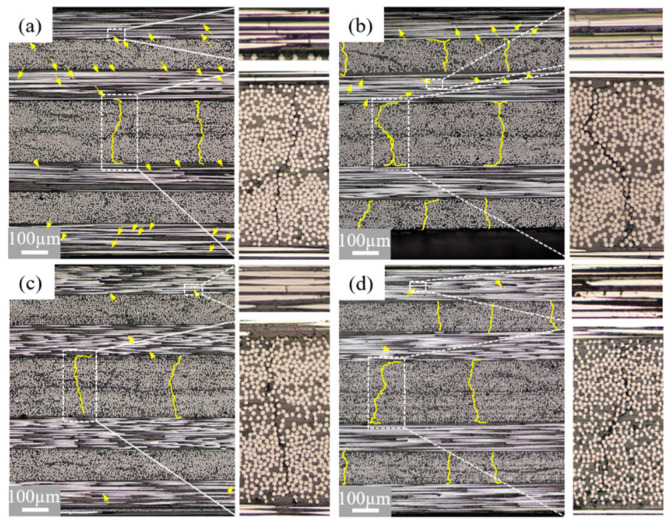
Damage images of the [0/75]_2s_ and [0/90]_2s_ laminates: (**a**) [0/75]_2s_ at the free edge; (**b**) [0/90]_2s_ at the free edge; (**c**) [0/75]_2s_ in the central section; (**d**) [0/90]_2s_ in the central section.

Microscopic observations of the damage progression in the width direction of the specimens were carried out for the [0/75]_2s_ and [0/90]_2s_ specimens. Typical observation results of [0/75]_2s_ and [0/90]_2s_ specimens are presented in Figure 8. From Figure 8, comparing the damage images at the free edge, much more fiber breakages (marked by yellow arrows) are observed at the free edge (Figure 8a,b), but very few are observed in the central section (Figure 8c,d). These results demonstrate that fiber breakages are limited to the free edge of the tested specimens attributed to defects on the fibers of the 0° plies caused in the polishing process. Inversely, matrix cracks are observed both at the free edge and in the central section, indicating that similar matrix cracks occur not only at the free edge, but also in any section in the width direction. Besides, matrix cracks propagate along the off-axial orientation through the width in off-axis plies. In addition, delamination-like damage is observed at the crack tips of some matrix cracks. Similar observation results were obtained for the [0/90]_2s_ laminate, which are shown in Figure 8d.

## 4. Finite Element Analysis of [0/θ]_s_ Laminates Subjected to Tension

### 4.1. Finite Element Analysis (FEA)

The finite element method (FEM) is useful for exploring the initiation and growth of microscopic damage events. A 3D micromechanical model of multidirectional laminate has been developed [27,28,29]. In this study, this 3D micromechanical model is used for the strength analysis of the present [0/θ]_ns_ laminates.

**Figure 9:**
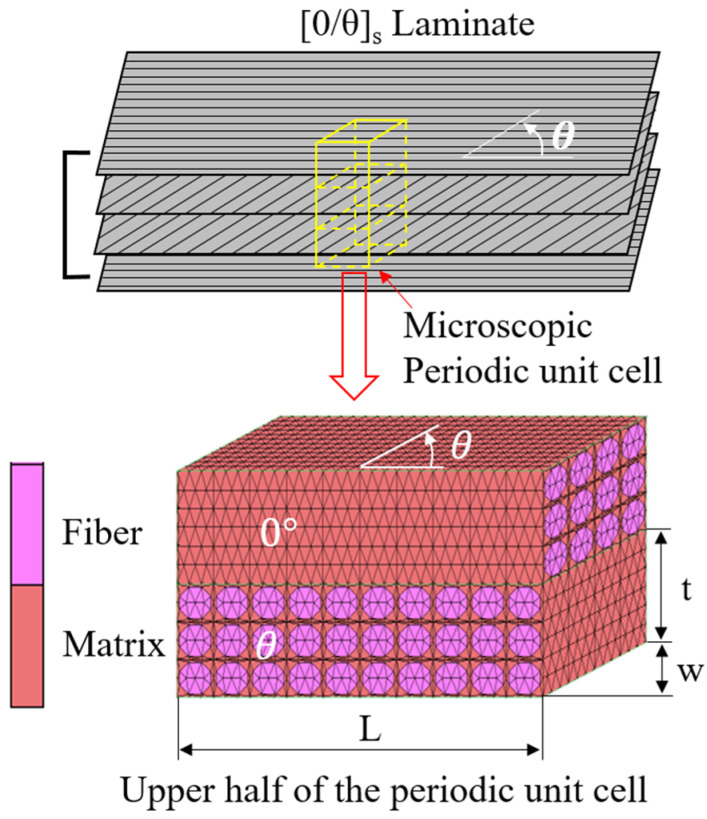
Periodic micromechanical model of a [0/θ]_s_ laminate.

Since it was expected that [0/θ]_s_ laminates possess similar mechanical properties to [0/θ]_2s_ laminates, and the present computational conditions did not allow us to simulate a full [0/θ]_2s_ laminate because of the limitation of the computer power, only the [0/θ]_s_ laminates were simulated. A micromechanical [0/θ]_s_ model is described in Figure 9. According to the symmetric stacking sequence of the tested laminate, only the upper half of the plies was simulated. Modeling matrix cracks always occurring in the off-axis layer and in the off-axis angle θ direction is more convenient, so a square fiber distribution in the [0/θ]_s_ laminates is assumed, as shown in Figure 9. Two sides of the in-plane parallelogram of the model were parallel to the *x*-axis and the other two sides of the in-plane parallelogram of the model were parallel to the off-axis angle θ in the off-axis layer (i.e., the off-axis angle θ direction) [30]. The thickness of one ply in the present model was 0.12 mm based on the experimental research. Moreover, it was difficult to exactly model more than 10 layers of fibers in one ply in the present computational condition. Thus, one ply of the present model contained only three fiber bundle layers. The fiber volume fraction was 58%. For all six [0/θ]_s_ laminates, their micromechanical models had the same thickness of 0.24 mm, and the same width in the y-direction, but different lengths in the x-direction, which varied from 0.618 to 0.4 mm, as the off-axis angle θ varied from 15° to 90°. According to the investigation of the effect of the element number on the computational results, all the micromechanical models of the six [0/θ]_s_ laminates had approximately 110,000 elements and the difference in the element numbers of the six models was less than 10%. A high order 10-node tetrahedral solid element was adopted in the nonlinear FEA to improve the analysis accuracy. The model generation and nonlinear FEA of the periodic micromechanical model of the [0/θ]_s_ laminates subjected to tension were carried out using MSC Marc 2011 [31].

In the present analysis, the carbon fibers are considered as transversely isotropic elastic material, and the matrix is considered as isotropic elastic-plastic material. The maximum stress criterion was adopted to simulate the fiber failure, and the maximum tensile strength is the stress at a failure strain of 2.0%. The material constants of the fiber and matrix are listed in Table 3. The relation of the tensile stress-equivalent plastic strain of the matrix is prescribed by a power law, and the relation is shown as follows:

(6) $$\sigma_{m}=A{\left(\varepsilon_{m}^{p}\right)}^{r}+\sigma_{y}$$

where *A* = 256, r = 0.259, and$\;\sigma_{y}$ = 30 (MPa), which were employed in the present study.

**Figure 10:**
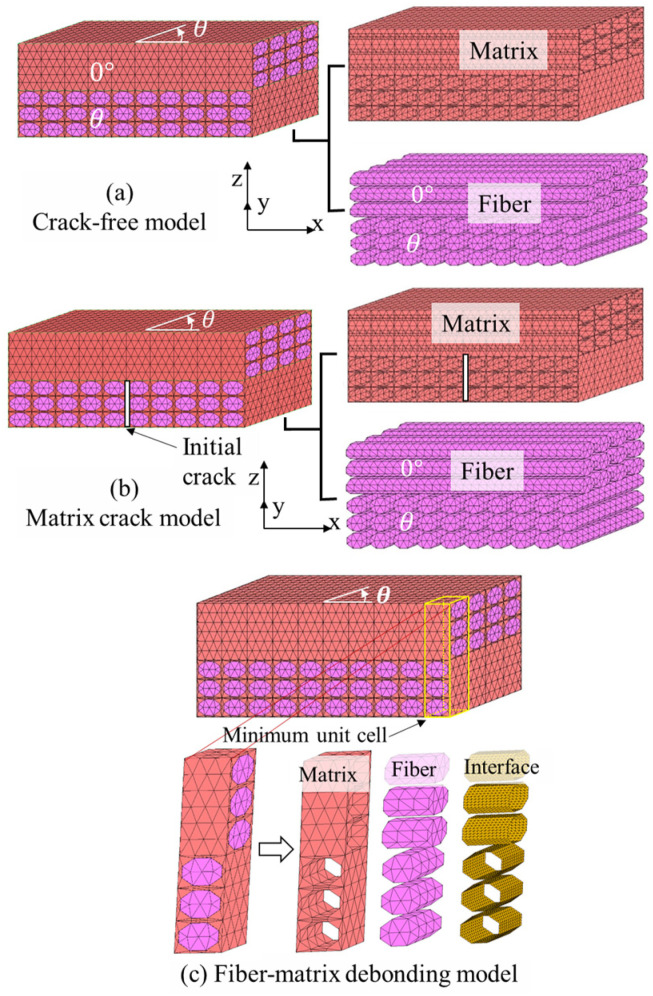
Three analysis models: (**a**) Crack-free model; (**b**) Matrix crack model with interface elements or initial crack inside; (**c**) Fiber-matrix debonding model.

Three micromechanical models, namely, the crack-free model, matrix crack model [28,32,33], and fiber-matrix debonding model, were analyzed to explore the failure mechanism of the [0/θ]_s_ laminates, as illustrated in Figure 10. In the crack-free model shown in Figure 10a, matrix cracks are neglected. In the matrix crack model (Figure 10b), two cases of matrix cracking and initial cracks are considered. In the case of matrix cracking, cohesive interface elements of zero thickness are inserted at the central plane of the off-axis layer to evaluate potential matrix cracking in the [0/θ]_s_ laminates. The cohesive interface is based on the thickness, and the width of the off-axis layer is based on the off-axis ply. A bilinear cohesive model is utilized to demonstrate the cohesive properties [28,30,31,32,33]. Material constants of the cohesive elements to simulate cracking in the matrix (listed in Table 4) were adopted to simulate potential matrix cracking in off-axis plies. In the case of the initial crack model, a crack of the matrix pre-existed at the central location of the off-axis ply. By contrast, the fiber-matrix debonding model (shown in Figure 10c) was used for modeling the effect of the off-axis layer on the fiber-matrix debonding. In Figure 10c, it can be seen that a minimum periodic unit model was taken out from the ordinary periodic micromechanical model to save on computational cost for the sake of balancing the computational power, cost, and accuracy of the computational results. Cohesive interface elements were added at the location where the fiber bundle and surrounding matrix were situated. The material constants of the interface element added to the analysis of the fiber-matrix debonding model are listed in Table 4.

The periodic boundary conditions can be expressed as follows [34,35,36]:

(7) $$u_{i}^{x+}\left(x,\;y,\;z\right)-u_{i}^{x-}\left(x,\;y,\;z\right)=\delta_{1i}\;\left(i=x,\;y,\;z\right)$$

(8) $$u_{i}^{y+}\left(x,\;y,\;z\right)-u_{i}^{y-}\left(x,\;y,\;z\right)=\delta_{2i}\;\left(i=x,\;y,\;z\right)$$

where superscripts *x*+/*y*+ and *x*−/*y*− denote the two opposite and parallel surfaces whose normal direction is along the *x*/*y*-direction, and $u_{i}$ is the displacement components in the *i*-direction. $\delta_{1i}$ and $\delta_{2i}$ are the displacements differences among the pair points located on the corresponding surfaces in the periodic model, both of which are solved during the computing process. In the present analysis, a function called servo-links in MSC Marc Mentat 2011 [31] was applied to estimate the periodic boundary condition, as shown in Figure 10 (θ = 45°). The red lines indicate that the periodic boundary conditions are activated.

**Figure 11:**
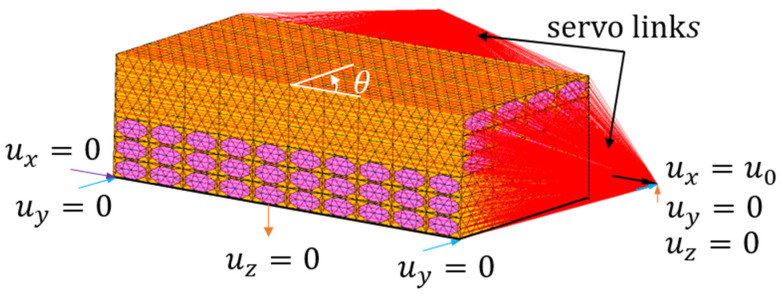
Boundary conditions applied to the micromechanical model.

Referring to the symmetry of the [0/θ]_s_ laminate, the following symmetrical boundary conditions

(9) $$u_{z}\left(x,y,0\right)=0,\;\;\tau_{zx}\left(x,y,0\right)=0,\;\;\tau_{yz}\left(x,y,0\right)=0$$

are applied to the bottom face of the model. The uniform tensile displacement $u_{x}=u_{0}$ is applied to the model with the aid of an unconstrained node, as shown in Figure 11. Nonlinear finite element analysis for all above-mentioned three micromechanical models is conducted based on a updated Lagrangian incremental method.

### 4.2. Numerical Results

**Figure 12:**
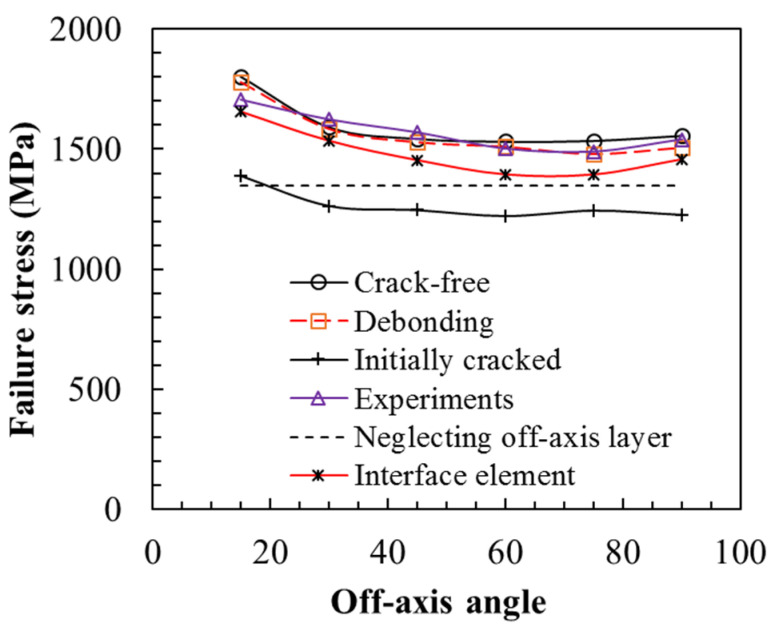
Numerical results of a series of FEA models compared to the experimental results.

The numerical results of the tensile failure strength of the [0/θ]_s_ laminates are presented in Figure 12 against the off-axis angle, together with the experimental results and a reference stress (dashed horizontal line), which neglects the contribution of the off-axis ply to the tensile properties of the laminate. Tensile strength is obviously reduced when the off-axis angle increases from 15° to 45° and exhibits a slight change as the off-axis angle θ becomes larger than 45°. The results obtained from the crack-free model and the fiber–matrix debonding model exhibit an agreement with the experimental results, revealing that a few matrix cracks occur during the tensile process.

The calculation results of the matrix crack model with the interface element are a little lower than the experimental ones. Since in the present analysis, interfacial constants may not be suitable for the real interfacial property in the laminate, it is necessary to obtain the actual critical energy release rate of the matrix crack based on the fracture toughness test of the laminate. Furthermore, the results obtained from the initial crack model are reduced by about 20%, and they are even lower than those for the referred strength, revealing that the off-axis layer with the initial matrix crack has a negative contribution to the strength of the [0/θ]_s_ plies. Thus, the analysis results obtained from the initial crack model indeed provide the lower bound of the strength of the [0/θ]_s_ plies.

All the results from the four cases are higher than the referred strength, revealing that off-axis ply still positively affects the tensile strength of the total plies. These results imply that matrix cracks occurring in the off-axis layer are affected by the tensile property of the whole plies.

**Figure 13:**
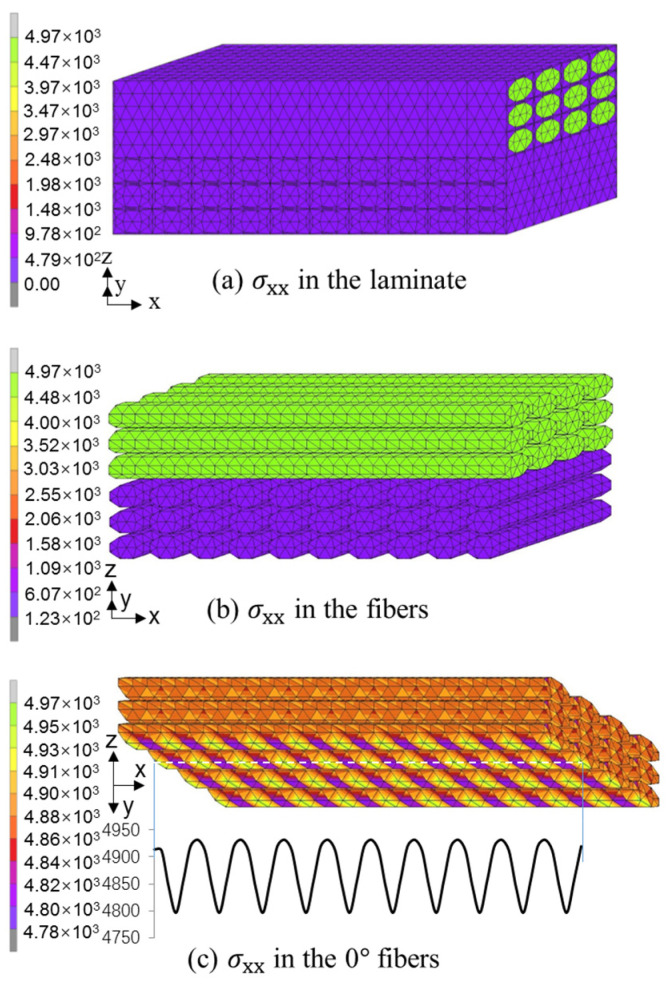
Tensile stress distribution in the [0/45]s laminate under failure stress obtained from the crack-free model: (**a**) σ_xx_ in the laminate; (**b**) σ_xx_ in the fibers; (**c**) σ_xx_ in the 0° fibers.

**Figure 14:**
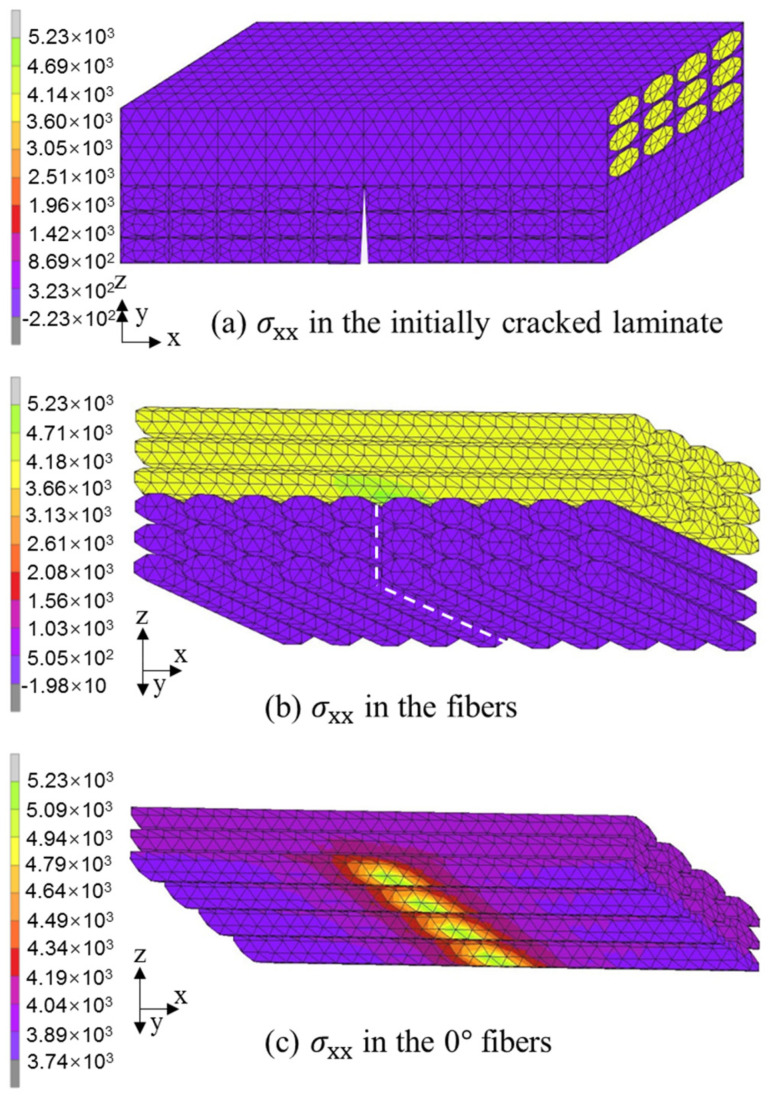
Tensile stress distribution in the [0/45]s laminate under failure stress obtained from the initial crack model: (**a**) σ_xx_ in the laminate; (**b**) σ_xx_ in the fibers; (**c**) σ_xx_ in the 0° fibers.

The stress distribution in the [0/θ]_s_ laminates was investigated using the crack-free model and initial crack model. All six [0/θ]_s_ laminates show a similar distribution, thus avoiding unnecessary duplication. As an example, only the analysis results for the [0/45]_s_ laminate are presented in Figure 13 and Figure 14.

In the case of the crack-free model, it is seen that the tensile stress in the fiber bundles of the 0° layer is much higher than that in the matrix and fiber bundles of the off-axis layer, as shown in Figure 13a,b. Furthermore, it is interesting that, as shown in Figure 13c, the tensile stress varies in the lower fiber layer in the 0° layer due to the effect of the fiber distribution in the off-axis layer, and the difference between the high and low stresses in the lower fiber layer of the 0° layer is about 100 MPa, which indicates that the [0/θ]_s_ laminate always fails in the off-axis angle of the off-axis layer. Therefore, due to the existence of fibers in the off-axis layers, there is a wave-like variation in the tensile stress in lower fiber bundles. This result is consistent with the experimental observation of the [0/θ]_2s_ laminates.

In the case of the initial crack model, the tensile stress in the fiber bundles of the 0° layer is also much higher than that in the matrix and fiber bundles of the off-axis layer, as shown in Figure 14a,b. The white dashed line in Figure 14b shows the location of the initial matrix crack. In Figure 14c, a very high tensile stress concentration is observed in the lower fiber layer of the 0° layer because of the effect of stress singularity at the crack tip. This result indicates that the initial matrix crack more easily induces unexpected defects in the [0/θ]_s_ laminate.

**Figure 15:**
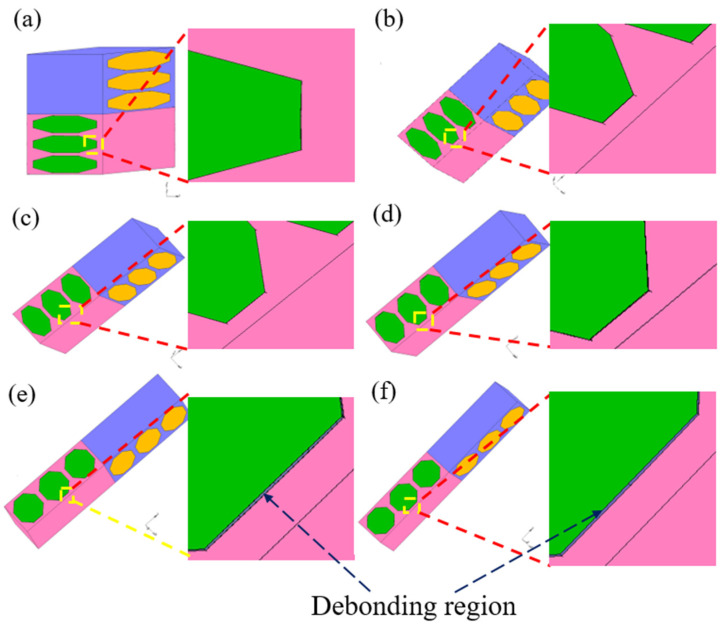
Interface deformation in various [0/θ]s laminates: (**a**) [0/15]s laminate; (**b**) [0/30]s laminate; (**c**) [0/45]s laminate; (**d**) [0/60]s laminate; (**e**) [0/75]s laminate; and (**f**) [0/90]s laminate.

In the case of the fiber-matrix debonding model, deformation of interface elements between fibers and matrix for various [0/θ]_s_ plies subjected to tension is presented in Figure 15. Each enlarged image for each [0/θ]_s_ laminate demonstrates the fiber-matrix interface location. In each case of an off-axis angle θ < 60°, a perfect bonding state between fiber and matrix is observed until failure. In the case of θ = 60°, the interface line perpendicular to the load direction is slightly thicker than in other cases of θ < 60°, implying that some damage occurs in the interface elements, although the fiber-matrix debonding crack is not formed yet. In the cases of the [0/75]_s_ and [0/90]_s_ laminates, clear debonding cracks are observed at the fiber–matrix interfaces, indicating that debonding has occurred at the fiber–matrix interface for both laminates. These results show a good consistency with the experimental ones.

**Figure 16:**
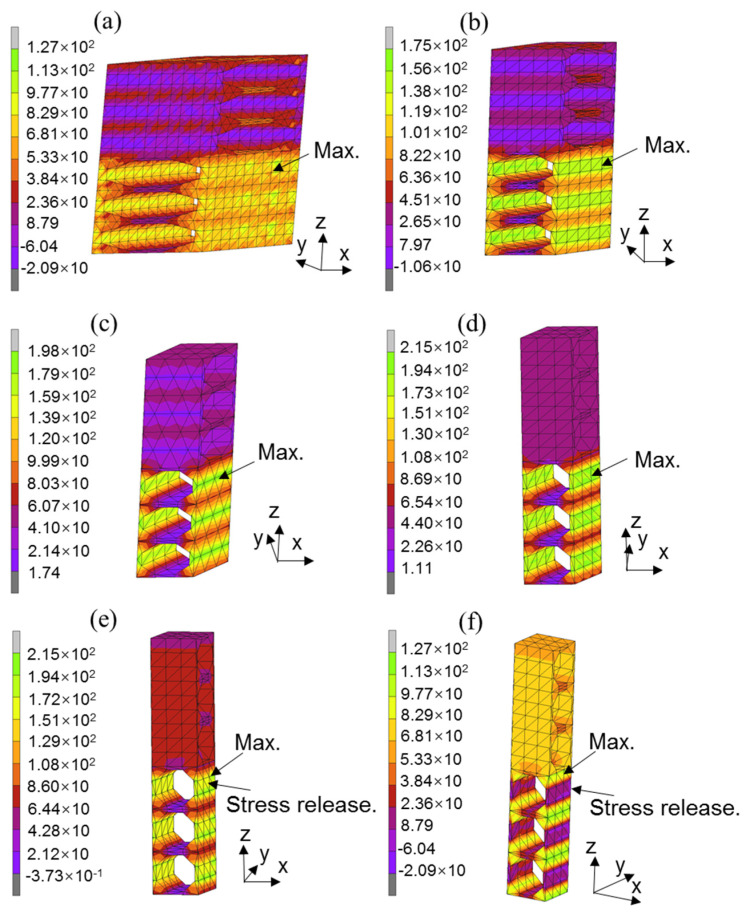
Stress distribution of the Von Mises stress in the [0/θ]s laminates: (**a**) [0/15]2s; (**b**) [0/30]2s; (**c**) [0/45]2s; (**d**) [0/60]2s; (**e**) [0/75]2s; and (**f**) [0/90]2s.

In Figure 16, the tensile stress distribution in the matrix under failure stress is presented for various [0/θ]_s_ laminates to demonstrate the effect of the off-axis angle on the major debonding stress at the fiber–matrix interface. In the four cases of [0/θ]_s_ laminates with an off-axis angle θ < 75, the maximum tensile stress is observed in the matrix of the off-axis layer in the central area of the side surface of each fiber layer, as indicated by arrows. The maximum tensile stress increases as the off-axis angle increases, indicating an increase of the riskiness of the fiber–matrix debonding in the off-axis layer with a large off-axis angle. In the cases of [0/75]_s_ and [0/90]_s_ laminates, different stress distributions are observed. In the case of the [0/75]_s_ laminate (Figure 16e), the maximum tensile stress has the same value as that of the [0/60]_s_ laminate, but the maximum tensile stress is distributed in both sides of the central area of the side surface of each fiber layer, and the tensile stress in the central area of the surfaces is lower than the maximum stress. These results indicate that a stress release occurs in the central area because of the initiation of a debonding crack in the central region of the fiber–matrix interface. In the case of the [0/90]_s_ laminate (Figure 16f), the maximum tensile stress is distributed in the areas outside of the central area, the value of the maximum tensile stress decreases sharply, and a very low tensile stress is observed in the central area of the side surface of each fiber layer, as indicated by arrows. These results imply that a full debonding crack is formed at the fiber–matrix interface adjacent to the central area of the side surface of each fiber layer, and a stress release occurs in this area.

## 5. Conclusions

The effect of the off-axis layer on tensile properties of unbalanced symmetric [0/θ]_2s_ laminate was investigated through experimental and numerical analysis. Six CFRP [0/θ]_2s_ laminates with θ = 15°, 30°, 45°, 60°, 75°, and 90° were fabricated for tensile tests. In situ observation of the progressive microscopic damage in the laminates was conducted. Finite element analysis of the [0/θ]_s_ laminate was conducted using a 3D micromechanical model. Based on the experimental and finite element analyses, the following conclusions are drawn.

(1) All six [0/θ]_2s_ plies with an off-axis angle θ ranging from 15° to 90° show approximate linear stress–strain responses in the tensile tests. A simple theoretical approximate formula is proposed to evaluate the tensile strength using material constants of unidirectional lamina. The prediction results for the tensile strength of the [0/θ]_2s_ laminates agree well with the experimental results.
(2) Matrix cracks were not observed prior to the final catastrophic failure in off-axis layers of the [0/θ]_2s_ laminates with a θ in the range of 15°–60°. Multiple matrix cracks were observed in the [0/75°]_2s_ and [0/90°]_2s_ plies only as the tensile strength increased from 50% to 80%. Delamination was not observed prior to the final catastrophic failure in all the [0/θ]_2s_ laminates during the tensile tests.
(3) Numerical analysis based on the crack-free micromechanical model leads to an upper bound of the tensile strength of the [0/θ]_s_ laminates. The micromechanical model with potential matrix cracking leads to similar results to those obtained from the crack-free model. The numerical results from the initial crack micromechanical model show a lower bound of tensile strength of the [0/θ]_s_ plies. A high stress concentration is observed adjacent to the cracked off-axis layer, inducing tensile strength loss of about 20%. Additionally, the simulated deformation results obtained from the fiber-matrix debonding model are consistent with the observation results from the tensile tests.

## Figures and Tables

**Table 1:** Stiffness constants of UD lamina.

E_1_ (GPa)	E_2_,E_3_ (GPa)	ν_12_, ν_13_	ν_23_	G_12_, G_13_ (GPa)	G_23_ (GPa)
134	9.5	0.34	0.48	3.18	3.22

**Table 2:** Experimental results of the tensile strength tests of the six [0/θ]_2s_ laminates.

Off-Axis Angle θ	Modulus (GPa)	C.V.	Failure Strain (%)	C.V.	Strength (MPa)	C.V.
15	99.60	0.042	1.91	0.036	1706.15	0.063
30	77.79	0.027	2.08	0.032	1610.58	0.042
45	71.08	0.038	2.08	0.030	1569.48	0.012
60	68.57	0.024	2.02	0.019	1501.32	0.015
75	69.91	0.012	2.01	0.038	1490.04	0.036
90	69.79	0.023	2.03	0.025	1520.65	0.022

**Table 3:** Material constants of the fiber and matrix.

Constants	Carbon Fiber (f)	Epoxy (e)
E_f11_, E_e11_ (GPa)	235	3.3
E_f22_, E_f33_, E_e22_, E_e33_ (GPa)	13	3.3
ν_f12_, ν_f13_, ν_e12_, ν_e13_	0.2	0.38
ν_f23_, ν_e23_	0.3	
G_f12_, G_f13_, G_e12_, G_e13_ (GPa)	15	1.2
G_f23_, G_e23_ (GPa)	5	

**Table 4:** Constants of cohesive elements inserted at different locations.

Items	Element inside Matrix	Elements at Fiber-Matrix Interface
Critical energy release rate (N/mm)	0.4	0.002
Critical opening displacement (mm)	6 × 10^−5^	8 × 10^−7^
Maximum opening displacement (mm)	0.01	5 × 10^−5^
Initial stiffness (N/mm^3^)	1.33 × 10^6^	10^8^
Critical traction (N/mm^2^)	80	80
